# Does Oral Health Status Predict Incident Frailty Over the 13‐Year Follow‐Up in Older Adults in England?

**DOI:** 10.1111/cdoe.70067

**Published:** 2026-03-31

**Authors:** Egle Petrauskiene, Paola Zaninotto, Anja Heilmann, Anne Peasey, Georgios Tsakos

**Affiliations:** ^1^ Department of Epidemiology and Public Health University College London London UK

**Keywords:** aged, frailty, longitudinal studies, oral health, quality of life

## Abstract

**Objectives:**

This study aimed to assess longitudinal associations between oral health and incident frailty, assessed by a multidimensional frailty index, in a national sample of older adults in England with participant mortality and drop out considered as competing risks.

**Methods:**

A nationally representative sample of adults aged ≥ 50 from the English Longitudinal Study of Ageing (ELSA) was analysed. Oral health was assessed using edentulousness, self‐rated oral health and the Oral Impacts on Daily Performances (OIDP) measures. Frailty was evaluated with the Rockwood frailty index. Competing risks regression models assessed the association between oral health at Wave 3 (2006/7) and incident frailty up to Wave 9 (2018/19), sequentially adjusting for demographic characteristics, socioeconomic factors and smoking, and accounting for participants' mortality and dropout.

**Results:**

Of 5760 non‐frail respondents at baseline, 1395 (24.2%) became frail during the 13‐years follow‐up. Edentate males had 1.40 (1.12**–**1.76) times higher risk of incident frailty than dentate males, whereas the association was not significant among females. Respondents with fair or poor self‐rated oral health had 1.48 (1.29**–**1.70) times and those that reported oral impacts had 1.85 (1.54**–**2.21) times higher risk of incident frailty than those reporting better oral health and no oral impacts respectively. Oral impacts on functional aspects (i.e., difficulties eating and speaking) were associated with increased incident frailty risk.

**Conclusions:**

Poor oral health can serve as a ‘canary in the coal mine’ and act as an early marker of frailty development among older adults in England. Future research should investigate whether public health strategies, such as oral health promotion and disease prevention, can help reduce the risk of frailty in later life.

## 1 Introduction

Oral conditions are the most prevalent chronic diseases globally and highly common in older individuals [1]. The combination of ageing societies and an epidemiological transition towards lower levels of edentulousness and greater retention of natural teeth is reshaping the oral health needs of older adults, with evidence suggesting increased treatment needs in some cohorts [2, 3]. Active dental caries and periodontal diseases are prevalent and also the main drivers of incremental tooth loss. Besides, dry mouth and oral cancer are also prevalent. Impaired oral health results in oral pain and problems eating, affecting the nutrition and quality of life of older people [4].

Evidence suggests that poor oral health is associated with higher levels of frailty [5]. Frailty is a major healthcare challenge and is defined as a status of vulnerability to adverse health outcomes which include functional decline, hospitalisation, disability, long‐term care and death [6, 7]. Longitudinal studies in Japan [8, 9, 10, 11, 12, 13], the US [14], Mexico [15, 16] and China [17, 18] have documented an association between worse oral health status and frailty among older adults using established composite frailty outcome measures. In the UK, a cross‐sectional study observed an association between difficulty eating and frailty in older adults aged 85 and over [19]. Another study of White British men aged 71 and over [14, 20] showed that worse oral health status including edentulousness, fewer teeth, dry mouth symptoms and cumulative oral health problems were linked to a higher risk of incident frailty. However, longitudinal research on the association between oral health and frailty has mainly focused on the frailty phenotype, that is, the physical dimension of frailty [9, 10, 11, 14, 15, 16, 20]. Since poor oral health is consistently linked to cognitive decline [21, 22, 23, 24, 25, 26, 27], it is important to look into the evidence between oral health and complex frailty measures that also include cognitive components.

In that respect, two longitudinal studies from Japan [8, 12, 13], reports from the Chinese Longitudinal Healthy Longevity Survey [17, 18] and one longitudinal study in England [28] have used a composite measure of frailty with a cognitive component. The first study [8] showed that reduced masticatory function was associated with the progression to pre‐frailty or frailty, evaluated by the Kihon frailty checklist. These associations might have been overestimated as the employed frailty checklist contained questions on eating difficulties. The second study demonstrated that ill‐fitting dentures [12, 13] and reduced chewing ability [12, 13] were associated with an increased frailty index, whereas each additional tooth was associated with a lower frailty index [13]. Among older Chinese adults [17, 18], tooth loss was associated with a higher risk of frailty [17, 18], and mortality [18], while a study in England [28] showed that participants with fewer teeth had increased frailty rates over a 4‐year period.

Moreover, participants' mortality and dropout may act as competing outcomes in longitudinal studies on oral health and incident frailty. Attrition due to mortality and non‐participation are linked to gerontological outcomes and, hence, cannot be safely assumed to represent noninformative censoring [29]. However, to our knowledge, the risk of bias due to mortality and dropout has not been addressed in the previous studies on oral health and frailty [8, 9, 10, 11, 12, 13, 14, 15, 16, 17, 18, 19, 20, 28]. Therefore, the aim of this study was to assess the longitudinal associations between oral health and incident frailty, assessed by a multidimensional frailty index, in a national sample of older adults in England over a 13‐year period with participant mortality and dropout considered as competing risks.

## 2 Methods

### 2.1 The English Longitudinal Study of Ageing

This study uses data from the English Longitudinal Study of Ageing (ELSA), a nationally representative cohort study of community‐dwelling adults aged 50 and over in England in the year 2002 [30]. The initial ELSA sample was selected from the households which took part in the Health Survey for England (HSE) in 1998, 1999 and 2001. All age‐eligible people in the household that responded to HSE and also conducted an ELSA interview became ELSA ‘core members’. The ELSA cohort was examined biannually, providing repeated data for the same individuals. For this study, data from Wave 3 (2006/7) to Wave 9 (2018/19) were analysed. Wave 3 was chosen as a baseline for this analysis as it was the first wave when oral health was assessed. Data were collected every 2 years through face‐to‐face Computer Assisted Personal Interviews and self‐completion questionnaires, and through nurse visits every 4 years. Ethical approval for the ELSA was granted by the London Multi‐Centre Research Ethics Committee and all individuals provided written informed consent. Further information on ELSA data and sampling details are available at http://www.elsa‐project.ac.uk/.

### 2.2 Exposure Variables

Three self‐reported oral health variables were collected in ELSA Wave 3. First, edentulousness (total tooth loss) is a dichotomous variable differentiating between participants who were dentate (had natural teeth) and those that were edentate (had no natural teeth). Second, self‐rated oral health was assessed through the question ‘Would you say that your dental health is?’, and answers were dichotomised into excellent, very good or good versus fair or poor. Third, the Oral Impacts on Daily Performances (OIDP) is a validated measure of oral health‐related quality of life for older people [31]. The impact of oral conditions on the quality of life in ELSA was assessed using a simplified version of the OIDP. Respondents were asked whether they had the following impacts on their daily life due to the condition of their teeth, mouth and/or dentures: (i) difficulty eating food; (ii) difficulty speaking clearly; (iii) problems smiling without embarrassment; (iv) problems with emotional stability, for example, becoming more easily upset than usual; and (v) problems enjoying the company of other people such as family, friends or neighbours. The response options to each OIDP item were yes or no. An aggregate binary variable determined the prevalence of oral impacts, that is, whether the respondent reported any of the aforementioned oral impacts versus none.

### 2.3 Outcome Variable

The Frailty index (FI) was constructed using the methodology proposed by Rockwood and colleagues [32], and followed the protocol previously used in ELSA [33]. The FI consisted of 56 variables including a variety of conditions and systems (Table S1). It incorporated questions on disability, mobility, self‐rated health status, chronic illnesses, cognitive function and depressive symptoms. An FI score ranging between 0 and 1 was constructed for each participant by summing up the number of deficits they experienced and dividing that by the total number of assessed items. Individuals who had at least 30 out of 56 deficits with non‐missing values in the dataset were included in the analysis as this number has been reported as sufficient to accurately predict adverse outcomes [32]. Respondents were considered frail if their FI score was greater than 0.25 [34].

### 2.4 Covariates

Based on previous studies, the following sociodemographic characteristics were considered as potential confounding factors and used as covariates in the analysis: age (in years); sex (male versus female); cohabitation status (married or cohabiting versus neither married nor cohabiting); wealth (quintiles of total non‐pension wealth, a composite measure of financial wealth, physical wealth including land, business worth and jewellery, as well as housing wealth); education (degree or equivalent, secondary qualification, or no qualifications); and current cigarette smoking (Yes/No). For descriptive purposes, age was categorised into 5‐year age groups (i.e., 50**–**54, 55**–**59, 60**–**64, 65**–**69, 70**–**74, 75**–**79 and 80+). In regression models, age was fitted as a mean‐centred continuous variable with the aim of reducing micro multicollinearity [35].

### 2.5 Data Analysis

To assess the association between baseline oral health (at Wave 3) and incident frailty during a period of up to 13 years of follow‐up (across Waves 4 to 9 of ELSA), an analytical sample of participants who were not frail at baseline was used. The eligibility criteria for inclusion in the analytical sample were applied in the following order: (i) Wave 3 core members; (ii) non‐frail at baseline; (iii) had follow‐up data required for survival analyses, namely, not lost between Waves 3 and 4; (iv) lived in the community and not in an institution; (v) had no missing data on exposure, outcome or covariate variables. The characteristics between participants who were included and those excluded from the analytical sample were compared using logistic regression.

The descriptive characteristics of the analytical sample are presented as percentages for categorical variables whereas mean and standard deviation are given for the continuous age variable. Sociodemographic and behavioural characteristics of the sample were cross‐tabulated by the oral health variables at baseline, using chi‐squared test and test for trend to assess bivariate associations. The incidence of frailty was calculated and differences in frailty incidence rates across categories of baseline oral health exposures and covariates were tested using the log‐rank test.

Competing risks regression, which refers to a special type of survival analysis, was used to explore associations between baseline oral health and incident frailty, while also accounting for mortality (mortality data were obtained from the National Health Service data registry) and drop‐out from the study. The Fine and Grey's proportional subhazards model [36] was employed. This is based on the subdistribution hazard or subhazard [37], which estimates failure of the main event of interest (i.e., frailty) while taking into account the risk of competing events. This model allows to check the robustness of the results to attrition which may introduce bias due to individuals non‐randomly dropping out of the study over time. The proportional subhazards assumption was verified by including interactions between a covariate and a log function of time for all model covariates. Survival time was calculated as the interval between the interview dates at baseline and the wave when participants either became frail (main outcome of interest) or were censored (last point of contact). The model was adjusted for baseline sociodemographic and behavioural covariates in sequential steps. Model 1 was the crude model, Model 2 adjusted for demographic factors (sex, age and cohabitation status), Model 3 additionally included socioeconomic position (total non‐pension wealth and education) and Model 4 also included smoking. The estimates for the risk of developing frailty in the competing risk regression models are presented as subhazard ratios (SHR) together with the respective 95% CI. Since increasing age and female sex are established risk factors for frailty [38], fully adjusted competing risk models were tested for interactions with age and sex. Interaction with edentulousness was also tested to understand whether associations between baseline self‐rated oral health and oral impacts, with incident frailty differed by dentition status. As it is plausible that poor oral health at baseline could reflect underlying or early frailty, we also conducted an additional sensitivity analysis, where the competing risks model was estimated after excluding participants with poor baseline self‐rated oral health status.

The baseline descriptive analyses were computed with Wave 3 cross‐sectional sampling weights in order to correct for non‐response and to ensure estimates were representative of the national population. However, longitudinal competing risk models were not weighted because ELSA longitudinal weights have only been developed for the subset of respondents who took part in all waves [39]. Therefore, using longitudinal weights was not appropriate for this analysis, as participants censored before Wave 9 would have been excluded from the analyses.

All analyses were performed using the statistical software STATA version SE 15.1.

## 3 Results

Out of 8810 Wave 3 core members, 5760 individuals were eligible for inclusion in the baseline analytical sample (Figure 1). Participants in the oldest age group (i.e., aged 80 years and over), women, smokers, those with lower educational attainment, lower wealth, not married or cohabiting, and those with worse oral health status were more likely to be excluded from the baseline analytical sample (Table S2). Out of the 5760 participants, after exclusion due to death, dropout, movement to institutions or missing data, a total of 3104 were followed up until Wave 9 (Figure 1). Table 1 shows that the sample consisted of 48.8% males, and the mean age was 64.4 (SD 9.7) years. With regards to oral health, 13.1% of the respondents were edentate at baseline, 15.6% reported fair or poor oral health status, and 6.3% experienced one or more oral impacts. In terms of frailty, 7.9% of participants were frail by Wave 4, and this steadily increased in subsequent waves, and doubled by Wave 9 (16.1%). Table 2 presents bivariate associations between oral health and covariates. There was a higher proportion of individuals with worse oral health (i.e., edentulism, fair or poor self‐rated oral health and reporting one or more oral impacts) among individuals in lower (compared to higher) education and wealth categories, as well as among smokers compared to non‐smokers.

**FIGURE 1:**
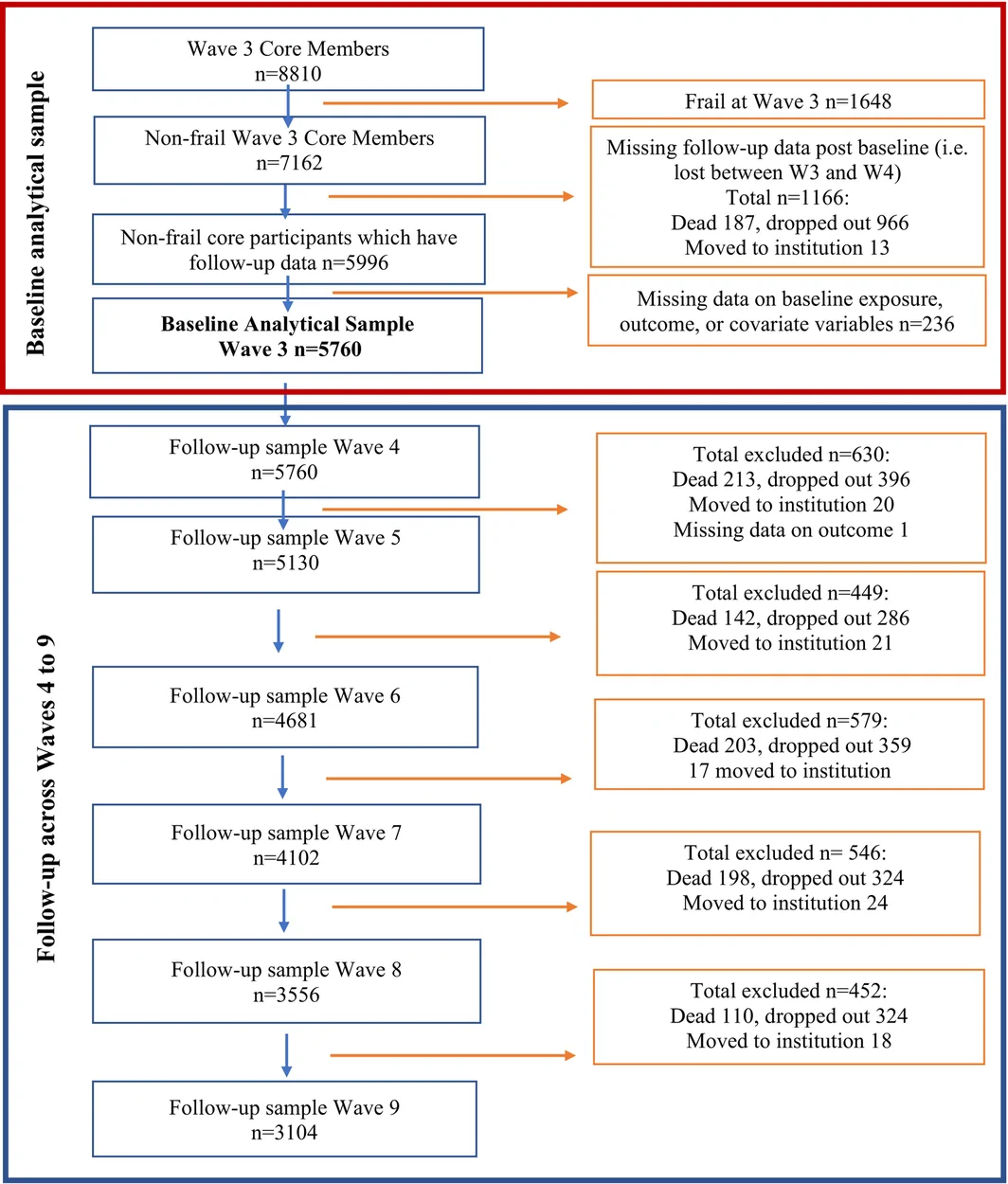
Flow chart of baseline and longitudinal analytical samples, ELSA.

**TABLE 1:** Baseline sample characteristics of non‐frail participants, ELSA wave 3 (2006–2007) and incident frailty across ELSA waves 4–9 (2008–2009 to 2018–2019) (*n* = 5760).

Characteristics	(%)^a^
Sex	
Male	48.8
Age (continuous): Mean (SD)	64.4 (9.7)
Cohabitation status	
Not married or cohabiting	27.6
Age groups	
50–54	14.8
55–59	23.3
60–64	18.1
65–69	14.6
70–74	11.5
75–79	10.6
80 and over	7.1
Education qualifications	
Degree or equivalent	42.3
Some qualifications	31.4
No qualification	26.3
Total non‐pension wealth (quintiles)	
1st (wealthiest)	24.5
2nd	22.5
3rd	20.8
4th	18.0
5th (poorest)	14.2
Current smoking status	
A smoker	13.7
Edentulousness	
Edentate	13.1
Self‐reported oral health status	
Fair or poor	15.6
Difficulty eating food	4.4
Reporting 1 or more oral impact(s)	6.3
Difficulty speaking clearly	0.8
Problems smiling without embarrassment	2.1
Problems with emotional stability	0.3
Problems enjoying the company of others	0.2
Incident frailty (score > 0.25)	
Wave 4 (2008–2009)—Frail (%)	7.9
Wave 5 (2010–2011)—Frail (%)	10.8
Wave 6 (2012–2013)—Frail (%)	11.7
Wave 7 (2014–2015)—Frail (%)	12.8
Wave 8 (2016–2017)—Frail (%)	14.2
Wave 9 (2018–2019)—Frail (%)	16.1

^a^ Weighted percentages and means.

**TABLE 2:** Sample characteristics according to oral health status of non‐frail participants. ELSA 2006–2007 (*n* = 5760).

Sociodemographic and behavioural characteristics	Oral health							
	Edentulousness	Self‐reported oral health	Oral impact(s)	Difficulty eating	Difficulty speaking	Problems smiling without embarrassment	Problems with emotional stability	Problems enjoying company of others
	Edentate (%)	Fair or poor (%)	Yes (%)	Yes (%)	Yes (%)	Yes (%)	Yes (%)	Yes (%)
Sex								
Male	11.6^a^	16.9^a^	7.0^a^	5.1^a^	1.0	1.9	0^a^	0.1
Female	14.4	14.3	5.7	3.4	0.6	2.3	0.4	0.3
Age groups								
50–54	3.5^d^	18.5^d^	6.9	5.0^c^	0.6	2.8^c^	0.6^c^	0.1
55–59	4.2	17.9	5.5	3.1	0.9	2.0	0.3	0.3
60–64	8.6	16.9	5.6	3.4	0.8	2.8	0.3	0.2
65–69	14.4	14.0	6.6	5.2	0.4	2.0	0.2	0
70–74	19.0	14.1	8.0	6.5	1.1	1.9	0.2	0.2
75–79	26.7	11.6	6.3	4.5	1.1	1.3	0	0.4
80 and over	40.8	9.9	6.5	5.0	0.9	1.1	0	0
Cohabitation status								
Married or cohabiting	10.4^b^	14.6^a^	6.2	4.3	0.9	2.0	0.2	0.2
Not married or cohabiting	20.0	18.0	6.7	4.7	0.7	2.5	0.4	0.3
Education qualifications								
Degree or equivalent	5.6^d^	14.6^d^	6.1	4.6	0.6	2.0	0.1	0.2
Some qualifications	11.5	14.7	6.2	3.8	0.9	2.4	0.4	0.3
No qualification	26.5	18.2	6.9	4.9	1.0	2.1	0.3	0.1
Total non‐pension wealth (quintiles)								
1st (wealthiest)	4.3^d^	11.8^d^	5.2^c^	3.8	0.6	1.4^c^	0.2	0
2nd	8.5	13.2	6.3	4.6	1.1	2.2	0.1	0.2
3rd	13.6	14.9	5.3	4.1	0.3	1.6	0	0
4th	18.1	19.2	7.3	4.3	1.4	2.6	0.6	0.5
5th (poorest)	28.2	22.1	8.7	5.9	0.8	3.5	0.3	0.2
Smoking								
Non‐smoker	11.9^b^	14.0^b^	5.7^b^	4.2^a^	0.7^a^	1.7^b^	0.2^a^	0.2^a^
A smoker	20.4	25.4	10.4	5.9	1.6	5.0	0.6	0.5

*Note:* Weighted percentages; *χ* ^2^ test for the difference between the two groups: ^a^ *p*‐value < 0.05, ^b^ *p*‐value < 0.001; test for trend: ^c^ *p*‐value < 0.05, ^d^ *p*‐value < 0.001.

Table 3 presents incidence of frailty by baseline oral health variables (Wave 3). During the study follow‐up (i.e., Waves 4 to 9), 1395 out of 5760 (24.2%) baseline respondents became frail. The follow‐up time ranged from 0.9 to 13 years, with an average of 8.4 years (SD 3.98). The overall incidence rate for frailty was 28.8 per 1000 person‐years. The incidence rate was significantly higher for edentate compared to dentate participants (66.6 vs. 24.7, *p* < 0.001), those who reported poor or fair oral health compared to better oral health ratings (38.7 vs. 27.1, *p* < 0.001), and those who reported one or more oral impacts compared to those without oral impacts (52.2 vs. 27.4, *p* < 0.001). The incidence of frailty during the follow‐up (Waves 4 to 9) by baseline (Wave 3) covariates is displayed in Table S3.

**TABLE 3:** Incidence of frailty by oral health status at baseline, ELSA 2006–2007 to 2018–2019 (*n* = 5760).

Oral health characteristics	Frailty incidence			
	Incident cases (*n*) (Total *n* = 1395)	Follow‐up (person‐years) (Total 48457.5 person‐years)	The incidence rate of frailty per 1000 person‐years (Total 28.8 per 1000 person‐years)	*p* ^a^
Edentulousness				
Dentate (*n* = 5027)	1078	43689.2	24.7	< 0.001
Edentate (*n* = 733)	317	4759.3	66.6	
Self‐reported oral health				
Excellent, very good, good (*n* = 4890)	1127	41526.8	27.1	< 0.001
Fair or poor (*n* = 870)	268	6930	38.7	
Oral impact(s)				
No (*n* = 5400)	1251	45701.2	27.4	< 0.001
Yes (*n* = 360)	144	2756.3	52.2	
Difficulty eating				
No (*n* = 5508)	1286	46539.8	27.6	< 0.001
Yes (*n* = 252)	109	1917.7	56.8	
Difficulty speaking				
No (*n* = 5714)	1374	48 146 0.8	28.5	< 0.001
Yes (*n* = 46)	21	310.7	67.6	
Problems smiling without embarrassment				
No (*n* = 5639)	1361	47491.2	28.7	0.256
Yes (*n* = 121)	34	966.2	35.2	
Problems with emotional stability				
No (*n* = 5745)	1391	48336.8	28.8	0.784
Yes (*n* = 15)	4	120.7	33.1	
Problems enjoying the company of others				
No (*n* = 5748)	1391	48373.2	28.8	0.302
Yes (*n* = 12)	4	84.3	47.4	

^a^ *p*‐value for log‐rank test for the differences in frailty incidence rates across the groups.

The results of the competing risks regression model estimating associations between baseline oral health variables and incident frailty across Waves 4 to 9 are presented in Table 4. There were significant interactions between edentulousness and sex, and between difficulty speaking clearly and edentulousness (Table S4), hence the corresponding models were stratified by sex and edentulousness, respectively. The proportional subhazards model assumptions were violated for total non‐pension wealth (in quintiles) in Model 3 for reporting at least one oral impact and difficulties eating, and Models 3 and 4 for difficulties speaking clearly among the edentate respondents. Therefore, total non‐pension wealth was fitted in binary form in models 3 and 4 for all oral health markers, which led to the model assumptions being met. The results of the models with the wealth covariate fitted as a binary variable (Table 4) were very close to those when wealth was entered in quintiles (Table S5).

**TABLE 4:** Oral health status and the risk of incident frailty from competing risks models. ELSA 2006–2007 to 2018–2019 (*n* = 5760).

Oral health characteristics	Competing risk model SHR (95% CI)			
	Model 1. Crude association	Model 2. Adjusted for sex, age and cohabitation status	Model 3. Adjusted for Model 2 and total non‐pension wealth (binary variable^d^) and education	Model 4. Adjusted for Model 3 and smoking
Edentulousness				
Males				
Dentate	1	1	1	1
Edentate	2.70 (2.21–3.29)^b^	1.72 (1.38–2.14)^b^	1.46 (1.16–1.82)^a^	1.40 (1.12–1.76)^a^
Females				
Dentate	1	1	1	1
Edentate	2.14 (1.82–2.52)^b^	1.45 (1.05–2.01)^a^, ^c^	1.10 (0.91–1.32)	1.07 (0.89–1.29)
Self‐reported Oral Health				
Excellent, v. good, good	1	1	1	1
Fair or poor	1.41 (1.23–1.61)^b^	1.57 (1.36–1.80)^b^	1.50 (1.31–1.73)^b^	1.48 (1.29–1.70)^b^
Oral impact(s)				
No	1	1	1	1
Yes	1.90 (1.60–2.25)^b^	1.95 (1.64–2.32)^b^	1.90 (1.59–2.27)^b^	1.85 (1.54–2.21)^b^
Difficulty eating				
No	1	1	1	1
Yes	2.08 (1.72–2.52)^b^	2.07 (1.70–2.52)^b^	2.5 (1.67–2.51)^b^	2.03 (1.65–2.48)^b^
Difficulty speaking				
Dentate				
No	1	1	1	1
Yes	2.12 (1.30–3.47)^a^	2.05 (1.22–3.43)^a^	1.91 (1.13–3.26)^a^	1.82 (1.06–3.12) ^a^
Edentate				
No	1	1	1	1
Yes	2.59 (1.17–5.75)^a^	3.50 (1.70–7.20)^a^	3.63 (1.70–7.75)^a^	3.45 (1.64–7.26)^a^
Problems smiling without embarrassment				
No	1	1	1	1
Yes	1.17 (0.84–1.63)	1.36 (0.97–1.92)	1.30 (0.93–1.83)	1.23 (0.87–1.73)
Problems with emotional stability				
No	1	1	1	1
Yes	1.14 (0.42–3.06)	1.38 (0.48–3.99)	1.15 (0.39–3.87)	1.13 (0.39–3.29)
Problems enjoying the company of others				
No	1	1	1	1
Yes	1.59 (0.54–4.68)	1.71 (0.55–5.26)	1.64 (0.55–4.94)	1.55 (0.52–4.63)

^a^ *p*‐value < 0.05.

^b^ *p*‐value < 0.001.

^c^ Proportional subhazard model assumptions were violated for cohabitation status (results after the removal of this covariate are displayed in Table S5).

^d^ Model assumptions were violated for total non‐pension wealth in quintile form in Model 3 for reporting at least on oral impact and difficulties eating, and Models 3 and 4 for difficulties speaking clearly among edentate. Therefore, this covariate was fitted in binary form in models 3 and 4 for all oral health markers, which led to the model assumptions being met. The results of the models with wealth covariate fitted as a binary variable (Table 4) were very close to those when wealth was entered in quintiles (Table S5).

The results of the competing risks regression model showed that unadjusted estimates (Table 4, Model 1) were significant for all three oral health variables, with the strongest association observed for edentulousness in males. Regarding specific oral impacts, difficulties eating and speaking were also associated with incident frailty (unadjusted model). Adjustment for sociodemographic factors and smoking (Models 2 and 3), overall, did not result in meaningful attenuations of the crude model estimates, apart from the associations between edentulousness and incident frailty in which adjustment for sociodemographic factors led to substantial attenuation of the effect sizes. In the fully adjusted competing risks model (Table 4, Model 4), there was a 40% increase in the risk of incident frailty in edentate compared to dentate respondents (SHR 1.40, 95% CI 1.12**–**1.75) among males, but the respective association was not significant among females (SHR 1.07, 95% CI 0.89**–**1.29). Older adults who reported poor or fair self‐rated oral health had a 1.48 (1.29**–**1.70) times higher risk of incident frailty compared to those who had better oral health ratings. Moreover, individuals who reported one or more oral impacts had 1.85 (1.54**–**2.21) times higher risk of incident frailty compared to their counterparts without oral impacts. In terms of specific oral impacts, there was a two‐fold increase in the risk of incident frailty among participants who had difficulties eating (SHR 2.03, 95% CI 1.65**–**2.48) in the fully adjusted model. There were also significant associations between incident frailty and difficulty speaking, and these were larger among the edentate (SHR 3.45, 95% CI 1.64**–**7.26) than the dentate (SHR 1.82, 95% CI 1.06**–**3.12) respondents. There was no significant association between incident frailty and the other oral impacts (Table 4).

The results of the sensitivity analysis, in which the competing risks model was re‐estimated after excluding participants with poor baseline self‐rated oral health status, are presented in Table S6. Overall, the estimates were very similar to those from the main analysis, with the exception the association between difficulty speaking and frailty among dentate participants, which was non‐significant with a slightly lower SHR and a wider 95% CI in the sensitivity analysis.

## 4 Discussion

This study documented that, in a large nationally representative sample of community‐dwelling adults aged 50 and over in England, poor oral health was associated with a higher risk of incident frailty. Edentulousness among men but not women, poor or fair self‐rated oral health, and reporting at least one oral impact were linked to a higher risk of frailty over the 13‐year follow‐up independently of sociodemographic factors, smoking, participant mortality and dropout. Of the specific oral impacts, those concerning functional aspects of oral health (i.e., difficulties eating and speaking) were associated with an increased risk of incident frailty. The findings of this study suggest that poor oral health can serve as a ‘canary in the coal mine’, and act as an early marker of frailty risk in older adults.

Previous longitudinal studies demonstrated that edentulousness [14, 16, 20], retaining fewer teeth [14, 15, 17, 18, 28], poor chewing ability [8, 12], difficulties eating [14], ill‐fitting dentures [12], dry mouth [14, 20] and cumulative oral health problems [11, 14, 20] were associated with higher risk of incident frailty, with follow‐up periods ranging between 1 and 16 years. Therefore, our study corroborates evidence on the long‐term influence of oral health on frailty development over a 13‐year period. To the best of our knowledge, this is also the first study on oral health to account for the competing risks of participant mortality and drop out, an important potential source of bias not considered in previous research. Moreover, previous longitudinal studies have mainly focused on a physical frailty outcome measure, using the Fried frailty phenotype [9, 10, 11, 14, 15, 16, 20]. The current study expands on existing evidence by showing that poor oral health is linked to a multidimensional frailty index including both physical and cognitive function elements. Future studies could further explore links between oral health and cognitive and physical domains of frailty.

This study also showed that the association between edentulousness and frailty was more pronounced in men. Previous studies [10, 18, 40] explored sex differences in the association between tooth loss and frailty. First, Hämäläinen and colleagues [40] showed that baseline grip strength correlated positively with number of teeth in men but not women, while no significant association between the number of teeth at baseline and change in grip strength over 5 years was observed in neither men nor women. Second, a 5‐year longitudinal study by Iwasaki and colleagues [10] that tested sex differences in the association between functional dentition and frailty phenotype did not observe significant moderation by sex. Third, in a study of older Chinese adults with a 16‐year follow‐up, no significant interactions by sex were reported in the association between dentition status and frailty index [18]. Compared to ELSA, these studies analysed samples that were considerably older at baseline (i.e., 80, 75 and 65 years old, respectively). Therefore, it is plausible that sex differences in the association between tooth loss and incident frailty may be less pronounced in older individuals.

Several pathways linking oral health and frailty are possible. First, there is evidence that associations between oral health and frailty may be mediated by nutritional status [17, 41, 42, 43, 44, 45]. Second, inflammation resulting from periodontal diseases may contribute to frailty development [5]. Previous research suggests that adjustment for inflammatory markers including IL‐6 [14] and CRP [46] attenuated the association between oral health and frailty. Among older Chinese adults, CRP explained ~5‐6% of the associations between dentition status and frailty [18]. Also, it has been hypothesised [5] that negative social consequences of poor oral health may link oral health and frailty. Since worse oral health is associated with loneliness and social isolation [47], which, in turn, are established risk factors for frailty [48], it is plausible that these act as potential mediators in the association between oral health and frailty; however, studies exploring this pathway are lacking. Moreover, it is possible that associations between oral health and frailty are bidirectional [5]. Frailty may contribute to worse oral health through less frequent dental attendance [49] and lack of adequate oral self‐care due to physical impairment [50].

The strengths of this study include the use of a large nationally representative sample of older adults aged 50 and over living in private households in England, which facilitates the generalisability of the findings. Also, a range of measures covering important aspects of older adults' oral health, frailty index components and the key confounding variables were used, in addition to adjustment for competing risks of participant mortality and dropout. A limitation of this study is that information on the number of natural teeth or denture use, important determinants of oral function, was not available for all these waves in ELSA. In addition, individuals with less favourable sociodemographic and oral health characteristics were more likely to be excluded from the baseline analytical sample. Therefore, it is possible that the associations between oral health and frailty may have been underestimated. We also acknowledge that it is plausible that baseline poor oral health status may reflect underlying but not yet documented frailty. We undertook sensitivity analysis excluding participants with poor self‐rated oral health at baseline, and the results confirmed the robustness of our findings, with only one exception for a specific oral impact with low prevalence at baseline (difficulty speaking). Also, given the complexity of frailty and its components, residual confounding, in particular arising from earlier life risk factors, cannot be ruled out. Future research should adopt a life course approach and assess the effect of early life risk factors, such as socioeconomic position and education [51], that may underpin links between oral health and frailty. Moreover, the hazard ratios from our survival models should be interpreted as reflecting the association between baseline oral health and subsequent incident frailty, under the simplifying assumption that oral health remains stable throughout the follow‐up period. We also acknowledge that while frailty is a dynamic and potentially reversible condition, in our study it was measured only at discrete waves; therefore limiting the ability to capture changes between assessments or to model frailty as a fully time‐varying state. Consequently, our reliance on baseline exposures and time‐to‐event models may not fully reflect temporal changes in either exposure or outcome. Future research using causal inference approaches, such as marginal structural models, could further explore time‐varying exposures and the dynamic relationship between oral health and frailty.

The findings of this study provide evidence that poor oral health can be considered an early marker of frailty risk in older people. Therefore, maintaining good oral health has potential to reduce the risk of frailty development and associated costs of care. People are affected by oral diseases across the life course, and these effects disproportionately accumulate in later life [52]. In England, with the decreasing rates of edentulousness and with more adults retaining their natural teeth into older age, there are increasing and complex dental treatment needs for the ageing cohorts [3, 53]. As such, public health initiatives including oral health promotion and disease prevention, particularly starting in earlier ages, are key to lower the burden of oral diseases in later life [52]. From a clinical and health service planning point of view, accessible and efficient preventive and treatment services are required across the lifecourse in order to prevent and treat oral diseases, which in turn may reduce the risk of frailty development in older ages.

In conclusion, this study provides longitudinal evidence from a nationally representative cohort study among older adults that individuals with poor oral health status have an increased risk of frailty. Future research should examine whether public health strategies, including oral health promotion and disease prevention, can reduce the risk of frailty later in life.

## Author Contributions

E.P., P.Z., A.H., A.P. and G.T. contributed to the study concept and design. E.P. conducted data analysis, and all authors contributed to the interpretation of the results. E.P. drafted the initial paper manuscript, and P.Z., A.H., A.P. and G.T. contributed to the critical revision of the content. All authors edited the final version of the manuscript. All authors have full access to all the data in the study, approved the final version of the manuscript, and have final responsibility for the decision to submit it for publication.

## Funding

The study was supported by the PhD studentship through the MRC grant MR/N013867/1. The English Longitudinal Study of Aging is funded by the National Institute on Ageing (Ref: R01AG017644) and by a consortium of UK government departments: Department for Health and Social Care; Department for Transport; Department for Work and Pensions, which is coordinated by the National Institute for Health Research (NIHR, Ref: 198–1074). Funding has also been provided by the Economic and Social Research Council (ESRC).

## Conflicts of Interest

The authors declare no conflicts of interest.

## Supporting information


**Table S1:** Variables used for the construction of the Frailty Index in ELSA 2006–2007 to 2018–2019.
**Table S2:** Comparison of participants that were included and excluded from the baseline analytical sample.
**Table S3:** Incidence of frailty by baseline covariates.
**Table S4:** Models with interaction terms between oral health factors, age and sex.
**Table S5:** Oral health status and the risk of incident frailty from competing risks models adjusted for quintiles of total non‐pension wealth.
**Table S6:** Oral health status and the risk of incident frailty from competing risks models.

## Data Availability

All waves of ELSA data are available from the UK Data Service https://beta.ukdataservice.ac.uk/datacatalogue/series/series?id=200011.

## References

[1] WHO , The Global Status Report on Oral Health 2022 [Internet] (2022), https://www.who.int/team/noncommunicable‐diseases/global‐status‐report‐on‐oral‐health‐2022.

[2] W. M. Thomson , “Epidemiology of Oral Health Conditions in Older People,” Gerodontology 31, no. s1 (2014): 9–16. 10.1111/ger.12085 24446974

[3] D. A. White , G. Tsakos , N. B. Pitts , et al., “Adult Dental Health Survey 2009: Common Oral Health Conditions and Their Impact on the Population,” British Dental Journal 213, no. 11 (2012): 567–572. 10.1038/sj.bdj.2012.1088 23222333

[4] A. E. Gerritsen , P. F. Allen , D. J. Witter , E. M. Bronkhorst , and N. H. J. Creugers , “Tooth Loss and Oral Health‐Related Quality of Life: A Systematic Review and Meta‐Analysis,” Health and Quality of Life Outcomes 8 (2010): 126. 10.1186/1477-7525-8-126 PMC2992503 21050499

[5] F. F. Hakeem , E. Bernabé , and W. Sabbah , “Association Between Oral Health and Frailty: A Systematic Review of Longitudinal Studies,” Gerodontology 36, no. 3 (2019): 205–215. 10.1111/ger.12406 31025772

[6] A. Clegg , J. Young , S. Iliffe , M. O. Rikkert , and K. Rockwood , “Frailty in Elderly People,” Lancet 381, no. 9868 (2013): 752–762. 10.1016/S0140-6736(12)62167-9 PMC4098658 23395245

[7] L. P. Fried , C. M. Tangen , J. Walston , et al., “Frailty in Older Adults: Evidence for a Phenotype,” Journals of Gerontology. Series A, Biological Sciences and Medical Sciences 56, no. 3 (2001): M146–M156. 10.1093/gerona/56.3.m146 11253156

[8] Y. Horibe , T. Ueda , Y. Watanabe , et al., “A 2‐Year Longitudinal Study of the Relationship Between Masticatory Function and Progression to Frailty or Pre‐Frailty Among Community‐Dwelling Japanese Aged 65 and Older,” Journal of Oral Rehabilitation 45, no. 11 (2018): 864–870. 10.1111/joor.12700 30040144

[9] M. Iwasaki , A. Yoshihara , N. Sato , et al., “A 5‐Year Longitudinal Study of Association of Maximum Bite Force With Development of Frailty in Community‐Dwelling Older Adults,” Journal of Oral Rehabilitation 45, no. 1 (2018): 17–24. 10.1111/joor.12578 28965370

[10] M. Iwasaki , A. Yoshihara , M. Sato , et al., “Dentition Status and Frailty in Community‐Dwelling Older Adults: A 5‐Year Prospective Cohort Study,” Geriatrics & Gerontology International 18, no. 2 (2018): 256–262. 10.1111/ggi.13170 28944598

[11] T. Tanaka , K. Takahashi , H. Hirano , et al., “Oral Frailty as a Risk Factor for Physical Frailty and Mortality in Community‐Dwelling Elderly,” Journals of Gerontology. Series A, Biological Sciences and Medical Sciences [Internet] 73 (2017): 1661–1667, https://academic.oup.com/biomedgerontology/advance‐article/doi/10.1093/gerona/glx225/4638577. 10.1093/gerona/glx225 29161342

[12] R. C. Castrejón‐Pérez , S. A. Borges‐Yáñez , R. Ramírez‐Aldana , I. Nasu , and Y. Saito , “Complete Dentures Associated With Frailty Among Edentulous Older Japanese People: A Prospective Analysis,” Community Dentistry and Oral Epidemiology 52, no. 4 (2024): 572–580. 10.1111/cdoe.12956 38509032

[13] R. C. Castrejón‐Pérez , S. A. Borges‐Yáñez , R. Ramírez‐Aldana , I. Nasu , and Y. Saito , “Self‐Reported Oral Diseases and Their Association With the Frailty Index Among Older Japanese People: Four‐Year Follow‐Up,” Journal of Frailty & Aging 13, no. 3 (2024): 259–266. 10.14283/jfa.2024.10 39082771

[14] R. Kimble , A. O. Papacosta , L. T. Lennon , et al., “The Relationship of Oral Health With Progression of Physical Frailty Among Older Adults: A Longitudinal Study Composed of Two Cohorts of Older Adults From the United Kingdom and United States,” Journal of the American Medical Directors Association 24, no. 4 (2023): 468–474.e3. 10.1016/j.jamda.2022.11.022 PMC10398566 36584971

[15] R. C. Castrejón‐Pérez , A. Jiménez‐Corona , E. Bernabé , et al., “Oral Disease and 3‐Year Incidence of Frailty in Mexican Older Adults,” Journals of Gerontology. Series A, Biological Sciences and Medical Sciences 72, no. 7 (2017): 951–957. 10.1093/gerona/glw201 28329793

[16] L. B. Velázquez‐Olmedo , S. A. Borges‐Yáñez , P. Andrade Palos , C. García‐Peña , L. M. Gutiérrez‐Robledo , and S. Sánchez‐García , “Oral Health Condition and Development of Frailty Over a 12‐Month Period in Community‐Dwelling Older Adults,” BMC Oral Health 21 (2021): 355. 10.1186/s12903-021-01718-6 PMC8290629 34284766

[17] X. Xu , Y. Zhao , B. Wu , Y. Pei , and D. Gu , “Association Between Tooth Loss and Frailty Among Chinese Older Adults: The Mediating Role of Dietary Diversity,” BMC Geriatrics 23 (2023): 668. 10.1186/s12877-023-04355-6 PMC10583397 37848821

[18] S. Lin , Q. Yu , J. Li , and X. Yan , “Dentition Status and Risk of Frailty in Older Chinese People: A 16‐Year Prospective Cohort Study,” Archives of Gerontology and Geriatrics 121 (2024): 105367. 10.1016/j.archger.2024.105367 38367564

[19] V. Albani , K. Nishio , T. Ito , et al., “Associations of Poor Oral Health With Frailty and Physical Functioning in the Oldest Old: Results From Two Studies in England and Japan,” BMC Geriatrics 21 (2021): 187. 10.1186/s12877-021-02081-5 PMC7977173 33736595

[20] S. E. Ramsay , E. Papachristou , R. G. Watt , et al., “Influence of Poor Oral Health on Physical Frailty: A Population‐Based Cohort Study of Older British Men,” Journal of the American Geriatrics Society 66 (2017): 473–479. 10.1111/jgs.15175 PMC5887899 29266166

[21] M. R. Nangle , J. Riches , S. A. Grainger , N. Manchery , P. S. Sachdev , and J. D. Henry , “Oral Health and Cognitive Function in Older Adults: A Systematic Review,” Gerontology 65, no. 6 (2019): 659–672. 10.1159/000496730 30904915

[22] R. Stewart , U. Stenman , M. Hakeberg , C. Hägglin , D. Gustafson , and I. Skoog , “Associations Between Oral Health and Risk of Dementia in a 37‐Year Follow‐Up Study: The Prospective Population Study of Women in Gothenburg,” Journal of the American Geriatrics Society 63, no. 1 (2015): 100–105. 10.1111/jgs.13194 25597561

[23] R. Stewart , R. J. Weyant , M. E. Garcia , et al., “Adverse Oral Health and Cognitive Decline: The Health, Aging and Body Composition Study,” Journal of the American Geriatrics Society 61, no. 2 (2013): 177–184. 10.1111/jgs.12094 PMC3578234 23405916

[24] R. Stewart , W. Sabbah , G. Tsakos , F. D'aiuto , and R. G. Watt , “Oral Health and Cognitive Function in the Third National Health and Nutrition Examination Survey (Nhanes Iii),” Psychosomatic Medicine 70, no. 8 (2008): 936–941. 10.1097/PSY.0b013e3181870aec 18842752

[25] R. Stewart and V. Hirani , “Dental Health and Cognitive Impairment in an English National Survey Population,” Journal of the American Geriatrics Society 55, no. 9 (2007): 1410–1414. 10.1111/j.1532-5415.2007.01298.x 17767683

[26] G. Tsakos , R. G. Watt , P. L. Rouxel , C. de Oliveira , and P. Demakakos , “Tooth Loss Associated With Physical and Cognitive Decline in Older Adults,” Journal of the American Geriatrics Society 63, no. 1 (2015): 91–99. 10.1111/jgs.13190 25523131

[27] Y. Fei , S. Niu , X. Xi , et al., “Physical Frailty Intensifies the Positive Association of Oral Frailty With Poor Global Cognitive Function and Executive Function Among Older Adults Especially for Females: A Cross‐Sectional Study,” BMC Geriatrics 24, no. 1 (2024): 468. 10.1186/s12877-024-05056-4 PMC11134949 38811863

[28] T. Komiyama , J. E. Gallagher , and Y. Hattori , “Relationship Between Tooth Loss and Progression of Frailty: Findings From the English Longitudinal Study of Aging,” Archives of Gerontology and Geriatrics 127 (2024): 105572. 10.1016/j.archger.2024.105572 39003834

[29] T. E. Murphy , L. Han , H. G. Allore , P. N. Peduzzi , T. M. Gill , and H. Lin , “Treatment of Death in the Analysis of Longitudinal Studies of Gerontological Outcomes,” Journals of Gerontology. Series A, Biological Sciences and Medical Sciences 66A, no. 1 (2011): 109–114. 10.1093/gerona/glq188 PMC3011957 21030467

[30] A. Steptoe , E. Breeze , J. Banks , and J. Nazroo , “Cohort Profile: The English Longitudinal Study of Ageing,” International Journal of Epidemiology 42, no. 6 (2013): 1640–1648. 10.1093/ije/dys168 PMC3900867 23143611

[31] G. Tsakos , W. Marcenes , and A. Sheiham , “Evaluation of a Modified Version of the Index of Oral Impacts on Daily Performances (OIDP) in Elderly Populations in Two European Countries,” Gerodontology 18, no. 2 (2001): 121–130. 10.1111/j.1741-2358.2001.00121.x 11794738

[32] S. D. Searle , A. Mitnitski , E. A. Gahbauer , T. M. Gill , and K. Rockwood , “A Standard Procedure for Creating a Frailty Index,” BMC Geriatrics 8 (2008): 24. 10.1186/1471-2318-8-24 PMC2573877 18826625

[33] N. T. Rogers , A. Marshall , C. H. Roberts , P. Demakakos , A. Steptoe , and S. Scholes , “Physical Activity and Trajectories of Frailty Among Older Adults: Evidence From the English Longitudinal Study of Ageing,” PLoS One 12, no. 2 (2017): e0170878. 10.1371/journal.pone.0170878 PMC5289530 28152084

[34] K. Rockwood and A. Mitnitski , “Frailty in Relation to the Accumulation of Deficits,” Journals of Gerontology. Series A, Biological Sciences and Medical Sciences 62, no. 7 (2007): 722–727. 10.1093/gerona/62.7.722 17634318

[35] D. Iacobucci , M. J. Schneider , D. L. Popovich , and G. A. Bakamitsos , “Mean Centering Helps Alleviate ‘Micro’ but Not ‘Macro’ Multicollinearity,” Behavior Research Methods 48, no. 4 (2016): 1308–1317. 10.3758/s13428-015-0624-x 26148824

[36] J. P. Fine and R. J. Gray , “A Proportional Hazards Model for the Subdistribution of a Competing Risk,” Journal of the American Statistical Association 94, no. 446 (1999): 496–509.

[37] R. J. Gray , “A Class of K‐Sample Tests for Comparing the Cumulative Incidence of a Competing Risk,” Annals of Statistics 16, no. 3 (1988): 1141–1154.

[38] A. Etman , A. Burdorf , T. J. M. V. der Cammen , J. P. Mackenbach , and F. J. V. Lenthe , “Socio‐Demographic Determinants of Worsening in Frailty Among Community‐Dwelling Older People in 11 European Countries,” Journal of Epidemiology and Community Health 66, no. 12 (2012): 1116–1121. 10.1136/jech-2011-200027 22544921

[39] NatCen Social Research , English Longitudinal Study of Ageing (ELSA). User Guide to the Main Interview Datasets Waves 1 to 9 (Natcen Social Research, 2020).

[40] P. Hämäläinen , T. Rantanen , M. Keskinen , and J. H. Meurman , “Oral Health Status and Change in Handgrip Strength Over a 5‐Year Period in 80‐Year‐Old People,” Gerodontology 21, no. 3 (2004): 155–160. 10.1111/j.1741-2358.2004.00022.x 15369018

[41] F. F. Hakeem , E. Bernabé , and W. Sabbah , “Association Between Oral Health and Frailty Among American Older Adults,” Journal of the American Medical Directors Association 22, no. 3 (2021): 559–563.e2. 10.1016/j.jamda.2020.07.023 32859517

[42] C. Bassim , A. J. Mayhew , J. Ma , et al., “Oral Health, Diet, and Frailty at Baseline of the Canadian Longitudinal Study on Aging,” Journal of the American Geriatrics Society 68, no. 5 (2020): 959–966. 10.1111/jgs.16377 32162690

[43] F. F. Hakeem , E. Bernabé , H. T. Fadel , and W. Sabbah , “Association Between Oral Health and Frailty Among Older Adults in Madinah, Saudi Arabia: A Cross‐Sectional Study,” Journal of Nutrition, Health & Aging 24, no. 9 (2020): 975–980. 10.1007/s12603-020-1506-1 PMC12280555 33155624

[44] S. Nakagawa , K. Miura , E. Arai , et al., “Oral Frailty, Appetite and Dietary Variety in Late‐Stage Older Adults: A Cross‐Sectional Study (The STudy of lAte‐Stage oldeR adulTs in Tottori; START Tottori),” Geriatrics & Gerontology International 24, no. 6 (2024): 626–633. 10.1111/ggi.14892 38714504

[45] X. Xia , Z. Yang , Z. Xu , et al., “Nutrition Status Plays a Partial Mediation Role in the Relationship Between Number of Teeth and Frailty: A Cross‐Sectional Multicenter Study,” Gerontology 70, no. 6 (2024): 572–584. 10.1159/000538181 38461811

[46] A. K. Welmer , D. Rizzuto , M. G. Parker , and W. Xu , “Impact of Tooth Loss on Walking Speed Decline Over Time in Older Adults: A Population‐Based Cohort Study,” Aging Clinical and Experimental Research 29, no. 4 (2017): 793–800. 10.1007/s40520-016-0630-6 PMC5533810 27682433

[47] A. Hajek , B. Kretzler , and H. H. König , “Oral Health, Loneliness and Social Isolation. A Systematic Review and Meta‐Analysis,” Journal of Nutrition, Health & Aging 26, no. 7 (2022): 675–680. 10.1007/s12603-022-1806-8 PMC9166168 35842757

[48] C. R. Gale , L. Westbury , and C. Cooper , “Social Isolation and Loneliness as Risk Factors for the Progression of Frailty: The English Longitudinal Study of Ageing,” Age and Ageing 47, no. 3 (2018): 392–397. 10.1093/ageing/afx188 PMC5920346 29309502

[49] B. Janssens , G. Tsakos , L. De Visschere , D. Verté , and N. De Witte , “Frailty as a Determinant of Dental Attendance Among Community‐Dwelling Older Adults,” Gerodontology 40 (2022): 363–371. 10.1111/ger.12664 36336964

[50] M. I. MacEntee , F. Müller , and C. Wyatt , Oral Healthcare and the Frail Elder: A Clinical Perspective [Internet] (Wiley‐Blackwell, 2010), https://www.wiley.com/en‐us/Oral+Healthcare+and+the+Frail+Elder%3A+A+Clinical+Perspective‐p‐9780813812649.

[51] W. M. Thomson and Y. Barak , “Tooth Loss and Dementia: A Critical Examination,” Journal of Dental Research 100, no. 3 (2021): 226–231. 10.1177/0022034520957233 32942945

[52] H. Benzian , C. C. Guarnizo‐Herreño , C. Kearns , M. W. Muriithi , and R. G. Watt , “The WHO Global Strategy for Oral Health: An Opportunity for Bold Action,” Lancet 398, no. 10296 (2021): 192–194. 10.1016/S0140-6736(21)01404-5 34174182

[53] J. Steele , E. Rooney , J. Clarke , and T. Wilson , “An Independent Review of NHS Dental Services in England [Internet],” (2009), https://www.google.com/search?q=Professor+J+Steele%2C+An+Independent+Review+of+NHS+Dental+Services+in+England%2C+2009&rlz=1C1ONGR_en‐GBGB981GB981&oq=Professor+J+Steele%2C+An+Independent+Review+of+NHS+Dental+Services+in+England%2C+2009&aqs=chrome..69i57.3712j0j7&sourceid=chrome&ie=UTF‐8.

