# Supplementary material for: Does Oral Health Status Predict Incident Frailty Over the 13‐Year Follow‐Up in Older Adults in England?
Source: Community Dent Oral Epidemiol. 2026 Mar 31;54(5):556–67. doi: 10.1111/cdoe.70067 (PMC13669801; doi:10.1111/cdoe.70067)
Supplement: Supplementary file 1 [file CDOE-54-556-s001.docx]

**Supplemental Material**

*Contents*

**Supplementary Table S1.** Variables used for the construction of the Frailty Index in ELSA 2006–2007 to 2018-2019

**Supplementary Table S2.** Comparison of participants that were included and excluded from the baseline analytical sample. ELSA, 2006–2007. n=8810

Supplementary Table S3. Incidence of frailty by baseline covariates. ELSA 2006–2007 to 2018–2019. n=5760

Supplementary Table S4. Models with interaction terms between oral health factors, age and sex. Competing risks model for attrition and mortality. ELSA 2006–2007 to 2018–2019. n=5760

Supplementary Table S5. Oral health status and the risk of incident frailty from competing risks models adjusted for quintiles of total non-pension wealth. ELSA 2006–2007 to 2018–2019. n=5760

**Supplementary Table S6**. Oral health status and the risk of incident frailty from competing risks models. Sensitivity analyses after excluding participants with poor baseline self-rated oral health status. ELSA 2006–2007 to 2018–2019. n=5591

**Supplementary Table S1. Variables used for the construction of the Frailty Index in ELSA 2006–2007 to 2018-2019.**

| **Frailty component variables** | **Categories and corresponding values assigned for the construction of the Frailty Index** |
| --- | --- |
| **Disability (experiencing ADL and iADL)** |  |
| Dressing, including putting on shoes and socks | No = 0; Yes = 1 |
| Walking across a room | No = 0; Yes = 1 |
| Bathing or taking a shower | No = 0; Yes = 1 |
| Eating, such as cutting up food | No = 0; Yes = 1 |
| Getting on and out of bed | No = 0; Yes = 1 |
| Using the toilet, including getting up or down | No = 0; Yes = 1 |
| Using a map to figure out how to get around a strange place | No = 0; Yes = 1 |
| Preparing hot meal | No = 0; Yes = 1 |
| Shopping for groceries | No = 0; Yes = 1 |
| Making telephone calls | No = 0; Yes = 1 |
| Taking medications | No = 0; Yes = 1 |
| Doing work around house and garden | No = 0; Yes = 1 |
| Managing money (e.g. paying bills, keeping track of expenses) | No = 0; Yes = 1 |
| **Experiencing Mobility Difficulties** |  |
| Walking 100 yards | No = 0; Yes = 1 |
| Sitting for about two hours | No = 0; Yes = 1 |
| Getting up from a chair after sitting for long periods | No = 0; Yes = 1 |
| Climbing several flights of stairs without resting | No = 0; Yes = 1 |
| Climbing one flight of stairs without resting | No = 0; Yes = 1 |
| Stooping, kneeling, or crouching | No = 0; Yes = 1 |
| Reaching or extending arms above shoulder level | No = 0; Yes = 1 |
| Pulling or pushing large objects like a living room chair | No = 0; Yes = 1 |
| Lifting or carrying weights over 10 pounds, like a heavy bag | No = 0; Yes = 1 |
| Picking up a 5p coin from a table | No = 0; Yes = 1 |
| **General health** |  |
| Self-reported general health | Excellent = 0;  Very good = 0.25;  Good = 0.5;  Fair = 0.75;  Poor = 1 |
| **Depressive symptoms** |  |
| Respondent felt depressed much of the time during past week | No = 0; Yes = 1 |
| Respondent felt that everything they did during the past week was an effort | No = 0; Yes = 1 |
| Respondent felt that their sleep was restless during the past week | No = 0; Yes = 1 |
| Respondent was not happy much of the time during the past week | No = 0; Yes = 1 |
| Respondent felt lonely much of the time during the past week | No = 0; Yes = 1 |
| Respondent did not enjoy life much of the time during the past week | No = 0; Yes = 1 |
| Respondent felt sad much of the time during the past week | No = 0; Yes = 1 |
| Respondent could not get going much of the time during the past week | No = 0; Yes = 1 |

| **Supplementary Table S1 (continued). Variables used for the construction of the Frailty Index in** **ELSA 2006–2007 to 2018-2019.** | |  |  |  |
| --- | --- | --- | --- | --- |
| **Frailty component variables** | **Cut off points and corresponding values assigned for the construction of the Frailty Index** | |  |  |
| **Self–reported conditions** |  | |  |  |
| High blood pressure | No = 0; Yes = 1 | |  |  |
| Angina | No = 0; Yes = 1 | |  |  |
| Myocardial infarction | No = 0; Yes = 1 | |  |  |
| Congestive heart failure | No = 0; Yes = 1 | |  |  |
| An abnormal heart rhythm | No = 0; Yes = 1 | |  |  |
| Diabetes or high blood sugar | No = 0; Yes = 1 | |  |  |
| A stroke (cerebral vascular disease) | No = 0; Yes = 1 | |  |  |
| Chronic lung disease | No = 0; Yes = 1 | |  |  |
| Asthma | No = 0; Yes = 1 | |  |  |
| Arthritis | No = 0; Yes = 1 | |  |  |
| Osteoporosis | No = 0; Yes = 1 | |  |  |
| Cancer | No = 0; Yes = 1 | |  |  |
| Psychiatric disease | No = 0; Yes = 1 | |  |  |
| Eyesight (while using lenses, if appropriate) | Excellent = 0;  Very good = 0.2;  Good = 0.4;  Fair = 0.6;  Poor = 0.8;  Blind = 1 | |  |  |
| Hearing (while using a hearing aid, if appropriate) | Excellent = 0;  Very good = 0.25;  Good = 0.5;  Fair = 0.75;  Poor =1 | |  |  |
| **Cognitive function** |  | |  |  |
| Parkinson’s disease | No = 0; Yes = 1 | |  |  |
| Alzheimer’s disease | No = 0; Yes = 1 | |  |  |
| Dementia or memory impairment | No = 0; Yes = 1 | |  |  |
| Did not successfully identified today's date | No = 0; Yes = 1 | |  |  |
| Did not successfully identify today's month | No = 0; Yes = 1 | |  |  |
| Did not successfully identify today's year | No = 0; Yes = 1 | |  |  |
| Did not successfully identify the day of the week | No = 0; Yes = 1 | |  |  |
| Immediate word recall | 1^st^ Quintile = 0;  2^nd^ Quintile = 0.25;  3^rd^ Quintile = 0.5;  4^th^ Quintile = 0.75;  5^th^ Quintile (lowest) = 1 | |  |  |
| Delayed word recall | 1^st^ Quintile = 0;  2^nd^ Quintile = 0.25;  3^rd^ Quintile = 0.5;  4^th^ Quintile = 0.75;  5^th^ Quintile (lowest) = 1 | |  |  |

**Supplementary Table S2. Comparison of participants that were included and excluded from the baseline analytical sample. ELSA, 2006–2007. n=8810**

| **Characteristics** | **Part of the baseline analytical sample N (%)**  **Total n=5760** | **Participants excluded from the baseline analytical sample N (%)**  **Total n=3050** | **OR (95%CI)** |
| --- | --- | --- | --- |
| **Sex**  Male  Female  Total | 2676 (46.5)  3084 (53.5)  5760 | 1265 (41.5)  1785 (58.5)  3050 | 1  1.22 (1.12–1.34) ^a^ |
| **Age groups**  50-54  55–59  60–64  65–69  70–74  75–79  80 and over  Total | 875 (15.2)  1228 (21.3)  1025 (17.8)  831 (14.4)  745 (12.9)  650 (11.3)  406 (7.0)  5760 | 513 (16.8)  430 (14.1)  396 (13.0)  345 (11.3)  385 (12.6)  406 (13.3)  575 (18.9)  3050 | 1  0.60 (0.51–0.70) ^a^  0.66 (0.56–0.77) ^a^  0.71 (0.60–0.84) ^a^  0.88 (0.75–1.04)  1.07 (0.90–1.26)  2.42 (2.04–2.86) ^a^ |
| **Cohabitation status**  Married or cohabiting  Not married or cohabiting  Total | 4107 (71.3)  1653 (28.7)  5760 | 1844 (60.5)  1206 (39.5)  3050 | 1  1.62 (1.48–1.78) ^a^ |
| **Education qualifications**  Degree or equivalent  Some qualifications  No qualification  Total | 2575 (44.7)  1804 (31.3)  1381 (24.0)  5760 | 892 (29.3)  881 (28.9)  1272 (41.8)  3045 | 1  1.41 (1.26–1.58) ^a^  2.66 (2.39–2.96) ^a^ |
| **Total non-pension wealth (quintiles)**  1^st (poorest)^  2^nd^  3^rd^  4^th^  5^th (wealthiest)^  Total | 757 (13.1)  1012 (17.6)  1194 (20.7)  1319 (22.9)  1478 (25.7)  5760 | 824 (30.4)  645 (23.8)  520 (19.2)  405 (14.9)  317 (11.7)  2711 | 1  0.59 (0.51–0.67) ^a^  0.40 (0.34–0.46) ^a^  0.28 (0.24–0.33) ^a^  0.20 (0.17–0.23) ^a^ |
| **Current smoking status**  Non-smoker  A smoker  Total | 5010 (87.0)  750 (13.0)  5760 | 2488 (81.7)  559 (18.3)  3047 | 1  1.50 (1.33–1.69) ^a^ |
| ^a^ p–value <0.001; unweighted percentages and odds ratios | | | |
|  |  |  |  |
| **Supplementary Table S2 (Continued). Comparison of participants that were included and excluded from the baseline analytical sample, ELSA 2006–2007. n=8810** | | | |
| **Characteristics** | **Part of the baseline analytical sample N (%)**  **Total N=5760** | **Participants excluded from the baseline analytical sample N (%)**  **Total N=3050** | **OR (95%CI)** |
| **Edentulousness**  Dentate  Edentate  Total | 5027 (87.3)  733 (12.7)  5760 | 2260 (74.2)  785 (25.8)  3045 | 1  2.38 (2.13–2.67) ^b^ |
| **Self-reported oral health**  Excellent, very good, good  Fair or poor  Total | 4890 (84.9)  870 (15.1)  5760 | 2287 (75.1)  757 (24.9)  3044 | 1  1.86 (1.67–2.08) ^b^ |
| **Reporting oral impact(s)**  No  Yes  Total | 5400 (93.8)  360 (6.2)  5760 | 2500 (86.8)  380 (13.2)  2880 | 1  2.28 (1.96–2.65) ^b^ |
| **Difficulty eating**  No  Yes  Total | 5508 (95.6)  252 (4.4)  5760 | 2599 (90.2)  281 (9.8)  2880 | 1  2.36 (1.98–2.82) ^b^ |
| **Difficulty speaking**  No  Yes  Total | 5714 (99.2)  46 (0.8)  5760 | 2805 (97.4)  75 (2.6)  2880 | 1  3.32 (2.29–4.81) ^b^ |
| **Problems smiling without embarrassment**  No  Yes  Total | 5639 (97.9)  121 (2.1)  5760 | 2768 (96.1)  112 (3.9)  2880 | 1  1.89 (1.45–2.45) ^b^ |
| **Problems with emotional stability**  -No  -Yes  Total | 5745 (99.7)  15 (0.26)  5760 | 2856 (99.2)  24 (0.8)  2880 | 1  3.22 (1.69–6.14) ^b^ |
| **Problems enjoying the company of others**  No  Yes  Total | 5748 (99.8)  12 (0.2)  5760 | 2860 (99.3)  20 (0.7)  2880 | 1  3.35 (1.63–6.9) ^a^ |

^a^ p-value <0.05; ^b^ p–value <0.001; unweighted percentages and odds ratios

Supplementary Table S3. Incidence of frailty by baseline covariates. ELSA 2006–2007 to 2018–2019. n=5760.

|  | **Frailty Incidence** | | |  |
| --- | --- | --- | --- | --- |
| **Covariates** | **Incident cases (n)**  **(Total n=1395)** | **Follow-up (person-years)**  **(Total 48457.5 person-years)** | **The incidence rate of frailty per 1,000 person-years**  **(Total 28.8 per 1000 person-years)** | **P–value for log-rank test for the differences in frailty incidence rates across the groups** |
| **Sex**  Male  Female | 556  839 | 22794.7  25662.7 | 24.4  32.7 | <0.001 |
| **Age groups**  50–54  55–59  60–64  65–69  70–74  75-79  80 and over | 116  139  185  200  237  311  207 | 7776.6  11975.2  9544.8  7159.6  6024.2  4107.4  1869.7 | 14.9  11.6  19.4  27.9  39.3  75.7  110.7 | <0.001 |
| **Cohabitation status**  Married or cohabiting  Not married or cohabiting | 811  584 | 36098.4  12359.1 | 22.5  47.3 | <0.001 |
| **Education qualifications**  Degree or equivalent  Some qualifications  No qualification | 439  446  510 | 23737.8  14950.7  9769.0 | 18.4  29.8  52.2 | <0.001 |
| **Total non-pension wealth (quintiles)**  1^st^ (wealthiest)  2^nd^  3^rd^  4^th^  5^th^ (poorest) | 214  263  310  310  298 | 13802.9  11676.9  10056.0  7802.2  5119.4 | 15.5  22.5  30.8  39.7  58.2 | <0.001 |
| **Smoking**  Non-smoker  A smoker | 1175  220 | 42822.2  5635 | 27.4  39.0 | <0.001 |

Supplementary Table S4. Models with interaction terms between oral health factors, age and sex. Competing risks model for attrition and mortality. ELSA 2006–2007 to 2018–2019. n=5760.

| **Oral Health Characteristics** | **SHR (95%CI)** ^a^ | | | |
| --- | --- | --- | --- | --- |
|  | **Age (continuous)** | **Sex** | **Edentulousness** | |
| **Edentulousness** |  |  | |  |
| Dentate | 1 | 1 | |  |
| Edentate | 0.98 (0.97–0.99)^b^ | 0.73 (0.56–0.96) ^b^ | | N/A ^c^ |
| **Self-reported Oral Health** |  |  | |  |
| Excellent, v. good, good | 1 | 1 | | 1 |
| Fair or poor | 0.99 (0.97–1.00)^b^ | 0.89 (0.67–1.18) | | 1.22 (0.79–1.86) |
| **Reporting at least one oral health impacts** |  |  | |  |
| 0 | 1 | 1 | | 1 |
| 1/5 | 0.99 (0.97–1.01) | 0.92 (0.65–1.32) | | 0.89 (0.57–1.40) |
| **Difficulty eating food** |  |  | |  |
| No | 1 | 1 | | 1 |
| Yes | 0.99 (0.97–1.01) | 0.91 (0.61–1.35) | | 0.60 (0.36–1.02) |
| **Difficulty speaking clearly** |  |  | |  |
| No | 1 | 1 | | 1 |
| Yes | 0.98 (0.93–1.03) | 0.78 (0.30–2.06) | | 2.60 (1.09–6.19) ^b^ |
| **Problems smiling without embarrassment** |  |  | |  |
| No | 1 | 1 | | 1 |
| Yes | 1.00 (0.96–1.04) | 2.05 (0.97–4.36) | | 1.76 (0.76–4.11) |
| **Problems with emotional stability** |  |  | |  |
| No | 1 | 1 | | 1 |
| Yes | 0.89 (0.74–1.08) | 0.30 (0.02–5.45) | | 0.76 (0.06–10.29) |
| **Problems enjoying the company of others** |  |  | |  |
| No | 1 | 1 | | 1 |
| Yes | 1.14 (0.92–1.40) | 1.54 (0.13–18.65) | | 2.37 (0.19–29.77) |

^a^ Model adjusted for sex, age, cohabitation status, total non-pension wealth, education, and smoking;

^b^ p–value <0.05;

^c^ N/A (not applicable)

Supplementary Table S5. Oral health status and the risk of incident frailty from competing risks models adjusted for quintiles of total non-pension wealth. ELSA 2006–2007 to 2018–2019. n=5760.

| **Oral Health Characteristics** | | **Competing risk model**  **SHR (95% CI)** | | | |  |
| --- | --- | --- | --- | --- | --- | --- |
|  |  | **Model 1. Crude** | **Model 2. Adjusted for sex, age & cohabitation status** | **Model 3. Adjusted for Model 2 & total non-pension wealth (quintiles) & education** | **Model 4. Adjusted for Model 3 & smoking** |  |
| **Edentulousness** | **Males** |  |  |  |  | |
|  | Dentate | 1 | 1 | 1 | 1 | |
|  | Edentate | 2.70 (2.21–3.29) ^b^ | 1.72 (1.38–2.14) ^b^ | 1.45 (1.16–1.81) ^a^ | 1.40 (1.12–1.75) ^a^ | |
|  | **Females** |  |  |  |  | |
|  | Dentate | 1 | 1 | 1 | 1 | |
|  | Edentate | 2.14 (1.82–2.52) ^b^ | ^c^ 1.31 (1.10–1.57) ^a^ | 1.07 (0.88–1.29) | 1.04 (0.86–1.26) | |
| **Self-reported Oral Health** | |  |  |  |  | |
| Excellent, v. good, good | | 1 | 1 | 1 | 1 | |
| Fair or poor | | 1.41 (1.23–1.61) ^b^ | 1.57 (1.36–1.80) ^b^ | 1.50 (1.30–1.72) ^b^ | 1.47 (1.28–1.70) ^b^ | |
| **Reporting at least one oral impact** | |  |  |  |  | |
| 0 | | 1 | 1 | 1 | 1 | |
| 1/5 | | 1.90 (1.60–2.25) ^b^ | 1.95 (1.64–2.32) ^b^ | 1.90 (1.59–2.27) ^b^ | 1.85 (1.55–2.21) ^b^ | |
| **Difficulty eating food** | |  |  |  |  | |
| No | | 1 | 1 | 1 | 1 | |
| Yes | | 2.08 (1.72–2.52) ^b^ | 2.07 (1.70–2.52) ^b^ | 2.06 (1.68–2.52) ^b^ | 2.03 (1.66–2.49) ^b^ | |
| **Difficulty speaking clearly** | **Dentate** |  |  |  |  | |
|  | No | 1 | 1 | 1 | 1 | |
|  | Yes | 2.12 (1.30–3.47 ^a^ | 2.05 (1.22–3.43) ^a^ | 1.86 (1.10–3.15) ^a^ | 1.77 (1.03–3.05) ^a^ | |
|  | **Edentate** |  |  |  |  | |
|  | No | 1 | 1 | 1 | 1 | |
|  | Yes | 2.59 (1.17–5.75) ^a^ | 3.50 (1.70–7.20) ^a^ | 3.94 (1.82–8.50) ^b^ | 3.74 (1.77–7.93) ^a^ | |
| **Problems smiling without embarrassment** | |  |  |  |  | |
| No | | 1 | 1 | 1 | 1 | |
| Yes | | 1.17 (0.84–1.63) | 1.36 (0.97–1.92) | 1.28 (0.91–1.80) | 1.21 (0.86–1.71) | |
| **Problems with emotional stability** | |  |  |  |  | |
| No | | 1 | 1 | 1 | 1 | |
| Yes | | 1.14 (0.42–3.06) | 1.38 (0.48–3.99) | 1.15 (0.39–3.37) | 1.13 ( 0.39–3.30) | |
| **Problems enjoying the company of others** | |  |  |  |  | |
| No | | 1 | 1 | 1 | 1 | |
| Yes | | 1.59 (0.54–4.68) | 1.71 (0.55–5.26) | 1.63 (0.55–4.85) | 1.54 (0.52–4.58) | |

^a^ p–value <0.05;

^b^ p–value <0.001;

^c^ model assumptions were violated for cohabitation status, hence, this model is adjusted only for age

Supplementary Table S6. Oral health status and the risk of incident frailty from competing risks models. Sensitivity analyses after excluding participants with poor baseline self-rated oral health status. ELSA 2006–2007 to 2018–2019. n=5591

| **Oral Health Characteristics** | | **Competing risk model SHR (95% CI)** | | | |
| --- | --- | --- | --- | --- | --- |
|  |  | **Model 1. Crude association** | **Model 2. Adjusted for sex, age & cohabitation status** | **Model 3. Adjusted for Model 2 & total non-pension wealth (binary variable^d^) & education** | **Model 4. Adjusted for Model 3 & smoking** |
| **Edentulousness** | **Males** |  |  |  |  |
|  | Dentate | 1 | 1 | 1 | 1 |
|  | Edentate | 2.74 (2.24–3.35)^b^ | 1.73 (1.38–2.16) ^b^ | 1.45 (1.16–1.83)^a^ | 1.40 (1.12–1.76)^a^ |
|  | **Females** |  |  |  |  |
|  | Dentate | 1 | 1 | 1 | 1 |
|  | Edentate | 2.19 (1.86–2.58) ^b^ | ^c^1.30 (1.09–1.57) ^a^ | 1.08 (0.89–1.31) | 1.05 (0.87–1.28) |
| **Self-reported Oral Health** | |  |  |  |  |
| Excellent, v. good, good | | 1 | 1 | 1 | 1 |
| Fair | | 1.38 (1.19–1.60) ^b^ | 1.53 (1.32–1.79) ^b^ | 1.48 (1.27–1.72) ^b^ | 1.46 (1.25–1.70) ^b^ |
| **Oral impact(s)** | |  |  |  |  |
| No | | 1 | 1 | 1 | 1 |
| Yes | | 1.92 (1.60–2.31) ^b^ | 1.90 (1.57–2.29) ^b^ | 1.88 (1.55–2.27) ^b^ | 1.83 (1.51–2.22) ^b^ |
| **Difficulty eating** | |  |  |  |  |
| No | | 1 | 1 | 1 | 1 |
| Yes | | 2.02 (1.64–2.49) ^b^ | 1.92 (1.55–2.38) ^b^ | 1.94 (1.56–2.42)^b^ | 1.92 (1.53–2.39) ^b^ |
| **Difficulty speaking** | **Dentate** |  |  |  |  |
|  | No | 1 | 1 | 1 | 1 |
|  | Yes | 1.95 (1.10–3.48) ^a^ | 1.82 (0.99–3.34) | 1.68 (0.90–3.16) | 1.59 (0.83–3.02) |
|  | **Edentate** |  |  |  |  |
|  | No | 1 | 1 | 1 | 1 |
|  | Yes | 2.78 (0.98–7.85) | 3.69 (1.46–9.32) ^a^ | 4.41 (1.63–11.92) ^a^ | 4.33 (1.65–11.35) ^a^ |
| **Problems smiling without embarrassment** | |  |  |  |  |
| No  Yes | | 1  1.19 (0.82–1.74) | 1  1.35 (0.92–1.98) | 1  1.30 (0.89–1.89) | 1  1.23 (0.84–1.81) |
| **Problems with emotional stability** | |  |  |  |  |
| No | | 1 | 1 | 1 | 1 |
| Yes | | 1.34 (0.42–4.31) | 1.50 (0.40–5.57) | 1.28 (0.33–4.93) | 1.24 (0.33–4.67) |
| **Problems enjoying the company of others** | |  |  |  |  |
| No | | 1 | 1 | 1 | 1 |
| Yes | | 1.36 (0.29–6.36) | 1.30 (0.25–6.72) | 1.29 (0.25–6.65) | 1.19 (0.24–5.97) |

^a^ p-value <0.05;

^b^ p-value <0.001;

^c^ proportional subhazard model assumptions were violated for cohabitation status, therefore this covariate was removed from this model; ^d^ model assumptions were violated for total non-pension wealth in quintile form in Model 3 for reporting at least on oral impact and difficulties eating, and Models 3 and 4 for difficulties speaking clearly among edentate. Therefore, this covariate was fitted in binary form in models 3 and 4 for all oral health markers, which led to the model assumptions being met.
